# Nucleotide Excision Repair Is Not Induced in Human Embryonic Lung Fibroblasts Treated with Environmental Pollutants

**DOI:** 10.1371/journal.pone.0069197

**Published:** 2013-07-19

**Authors:** Pavel Rossner, Andrea Mrhalkova, Katerina Uhlirova, Milada Spatova, Andrea Rossnerova, Helena Libalova, Jana Schmuczerova, Alena Milcova, Jan Topinka, Radim J. Sram

**Affiliations:** 1 Department of Genetic Ecotoxicology, Institute of Experimental Medicine AS CR, Prague, Czech Republic; 2 Institute of Plant Molecular Biology, Biology Centre AS CR, Ceske Budejovice, Czech Republic; University of Giessen Lung Center, Germany

## Abstract

The cellular response to genotoxic treatment depends on the cell line used. Although tumor cell lines are widely used for genotoxicity tests, the interpretation of the results may be potentially hampered by changes in cellular processes caused by malignant transformation. In our study we used normal human embryonic lung fibroblasts (HEL12469 cells) and tested their response to treatment with benzo[a]pyrene (B[a]P) and extractable organic matter (EOM) from ambient air particles <2.5 µm (PM2.5) collected in two Czech cities differing in levels and sources of air pollution. We analyzed multiple endpoints associated with exposure to polycyclic aromatic hydrocarbons (PAHs) including the levels of bulky DNA adducts and the nucleotide excision repair (NER) response [expression of *XPE, XPC* and *XPA* genes on the level of mRNA and proteins, unscheduled DNA synthesis (UDS)]. EOMs were collected in the winter and summer of 2011 in two Czech cities with different levels and sources of air pollution. The effects of the studied compounds were analyzed in the presence (+S9) and absence (–S9) of the rat liver microsomal S9 fraction. The levels of bulky DNA adducts were highest after treatment with B[a]P, followed by winter EOMs; their induction by summer EOMs was weak. The induction of both mRNA and protein expression was observed, with the most pronounced effects after treatment with B[a]P (–S9); the response induced by EOMs from both cities and seasons was substantially weaker. The expression of DNA repair genes was not accompanied by the induction of UDS activity. In summary, our results indicate that the tested compounds induced low levels of DNA damage and affected the expression of NER genes; however, nucleotide excision repair was not induced.

## Introduction

Particulate matter (PM) is a ubiquitous air pollutant that originates in the environment either as primary particles emitted directly into the atmosphere, or as secondary particles formed as a result of chemical reactions between gaseous pollutants or reactions between gases and primary particles [1]. Human exposure to PM is associated with increased mortality, particularly due to cardiovascular and respiratory diseases, including cancer [2], [3]. PM of a smaller aerodynamic diameter (<2.5 µm; PM2.5) poses greater health risks than coarse particles because of their ability to penetrate and accumulate in the lungs. The chemical composition of PM varies depending on the sources of pollution, season, climate and other factors. The major components of PM are organic compounds including polycyclic aromatic hydrocarbons (PAHs), quinones, transition metals, reactive gases, biological material, and minerals [2]. The carcinogenic properties of PM2.5 are namely associated with the presence of known carcinogens, including PAHs, arsenic, chromium, and nickel, although other constituents of PM2.5 may also play a role in tumor induction by this mixture [4].

PAHs are products of incomplete combustion of organic material. In the environment they are usually found in complex mixtures adsorbed to PM. Upon entering the organism, PAHs are metabolized by the cytochrome P450 enzymes to reactive intermediates that may bind to DNA, resulting in PAH-DNA adduct formation [5]. Many PAHs are known carcinogens in laboratory animals and probable or possible carcinogens in humans (IARC Group 2A and 2B). Benzo[a]pyrene (B[a]P) is carcinogenic in humans (IARC Group 1) [6]. The carcinogenic properties of PAHs are associated with their ability to induce DNA adduct formation and mutations in oncogenes and/or tumor suppressor genes. The presence of PAH adducts in DNA results in conformational changes of the DNA molecule, which impede the progress of DNA repair enzymes resulting in the formation of mutations [7]. PAH-DNA adducts are repaired by the action of nucleotide excision repair (NER). NER is a complex repair system intended to remove bulky lesions such as those induced by UV-radiation and/or by various chemical agents in the DNA molecule.

Unscheduled DNA synthesis (UDS) is a commonly used method for the analysis of NER activity. Among other experiments, UDS was used to measure nucleotide incorporation after the treatment of cells *in vitro* with carcinogens [8]. The most frequently used methods of UDS analysis include ^3^H-thymidine and bromodeoxyuridine (BrdU) incorporation into DNA and the evaluation of the results either by autoradiography, liquid scintillation counting of radioactivity, or immunofluorescence detection using anti-BrdU antibody [9]. Recently, a new method to measure UDS based on 5-ethynyl-2′-deoxyuridine (EdU) incorporation and its detection by a fluorescent azide through a Cu(I)-catalyzed [3+2] cycloaddition reaction (“click” chemistry) has been developed [10]. Unlike ^3^H-thymidine-based techniques, this method does not use radioactivity, and in comparison with the BrdU assay it is significantly faster and more sensitive.

In our study we used normal human embryonic lung fibroblasts (HEL12469 cells) and tested their response to treatment with B[a]P and extractable organic matter (EOM) from PM2.5 collected in two Czech cities differing in levels and sources of air pollution. We studied multiple endpoints associated with the cellular response to PAH exposure: the levels of bulky DNA adducts, the expression of selected NER genes (*XPE*, *XPC* and *XPA*) on the level of mRNA and proteins and the activity of NER. To account for possible lower metabolic activity of the fibroblasts, we conducted all treatments in the presence and absence of the rat liver microsomal S9 fraction. We hypothesized that B[a]P as a model PAH will increase bulky DNA adduct levels and subsequently induce a DNA repair response. We expected the response to EOM treatment to be generally less pronounced, with stronger effects for mixtures collected in winter in a location with air heavy pollution. The presence of the microsomal S9 fraction should further increase the intensity of the cellular response.

## Materials and Methods

### PM2.5 Collection, Sampling Sites, EOM Extraction and Chemical Analysis

PM2.5 was collected by a HiVol 3000 air sampler (model ECO-HVS3000, Ecotech, Australia) on Pallflex filters T60A20 (20×25 cm) in two locations in the Czech Republic differing in the extent and sources of air pollution: Prague (a sampler was located outside the city center, with moderate traffic intensity) and Ostrava (a sampler was located in a heavily polluted industrial area). To account for the seasonal variability of air pollutants, the samples were collected in the winter and summer seasons of 2011, for 24 h each day for 4–5 weeks. Each filter was extracted with 60 ml of dichloromethane and 3 ml of cyclohexane for 3 h. The extracts (EOMs) from all filters were pooled and aliquots were used for chemical analysis and cell treatment. Quantitative chemical analysis of eight PAHs – (benz[*a*]anthracene, benzo[*a*]pyrene, benzo[*b*]fluoranthene, benzo[*g*,*h*,*i*]perylene, benzo[*k*]fluoranthene, chrysene, dibenzo[*a*,*h*]anthracene and indeno[1,2,3-*c*,*d*]pyrene) – was performed by HPLC with fluorimetric detection. For the *in vitro* experiments, EOM samples were evaporated to dryness under a stream of nitrogen and the residue redissolved in dimethylsulfoxide (DMSO). The stock solution of each EOM sample contained 50 mg of EOM/ml DMSO. EOMs were kept in the freezer at −80°C. The extraction of PM2.5 and chemical analyses were performed in the laboratories of the certified company ALS Czech Republic, Prague (EN ISO CSN IEC 17025).

### Cell Cultures, Growth Characteristics and Cytotoxicity

For all tests, human embryonic lung fibroblasts (HEL12469, ECACC, Salisbury, UK) were used. For propagation the cells were grown at 37°C in a humidified atmosphere containing 5% CO_2_ in 75 cm^2^ tissue culture flasks (TPP, Trasadingen, Switzerland). We used minimal essential medium EMEM supplemented with 10% fetal bovine serum (FBS), 2 mM glutamine, 1% non-essential amino acids, 0.2% sodium bicarbonate, 50 U/ml penicillin and 50 µg/ml streptomycin. After reaching confluency, the cells were treated with 0.25% trypsin/0.02% EDTA in PBS and used for individual applications. Based on our previous experience with B[a]P (Sigma-Aldrich, St. Louis, MO, USA) and EOM cell toxicity [11], we selected three concentrations of B[a]P and EOMs for the treatment of HEL12469 cells (B[a]P: 1, 10 and 25 µM; EOMs: 1, 10 and 25 µg/ml). All compounds were tested both in the presence and absence of the rat liver microsomal S9 fraction (Toxila, Pardubice, Czech Republic). The +S9 mixture contained 8 mM MgCl_2_, 33 mM KCl, 100 mM (PO_4_)^-III^, 5 mM glucose-6-phosphate, 5 mM NADP, and the S9 fraction (protein concentration: 1 mg/ml), pH 7.4; in the –S9 mixture the S9 fraction was replaced with water. Mixtures were prepared fresh before use and were added to the culture medium along with the test compounds; their volume in the culture medium did not exceed 1%.

Before using the compounds and the S9 fraction for cell treatment, we tested their cytotoxicity and possible negative effects on the growth characteristics of the cells by constructing growth curves and calculating the doubling time (T) of the cells according to the formula:
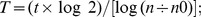

*t* = incubation time (h), *n* = number of cells at the end of incubation; *n0* =  initial number of cells.

The cytotoxicity of the tested compounds was analyzed using the cell proliferation reagent WST-1 (Roche, Mannheim, Germany) as described by the manufacturer. Briefly, the cells (1×10^5^) were seeded in a 96-well plate (TPP, Trasadingen, Switzerland) and grown for 2 days in 100 µl culture medium. The medium was then replaced with 100 µl fresh medium containing the test chemicals and S9 fraction. Incubation of the plate for 6 or 24 h at 37°C was followed by the addition of 10 µl WST-1 reagent and further incubation at 37°C for 30 min. Finally, the absorbance was read at 440 nm using a microplate reader, and the metabolic activity of the treated cells was determined by a comparison of the absorbances in the treated wells with those of the negative control (cells treated with DMSO only).

### DNA Adducts Analysis by ^32^P-postlabeling

The cells were grown and treated in the same way as samples for NER protein analysis. After harvesting, cell pellets were dissolved in a solution of 20 mM Tris–HCl, 10 mM EDTA and 1% sodium dodecyl sulfate (SDS), pH 8.0. DNA was isolated using RNAses A and T1 and proteinase K treatment followed by phenol/chloroform/isoamyl alcohol as previously described [12]. DNA concentration was estimated spectrophotometrically by measuring ultraviolet (UV) absorbance at 260 nm, while DNA purity was evaluated by measuring absorbance at 280 nm and calculating the 260/280 nm absorbance ratio. DNA samples were stored at −80°C until analysis.

^32^P-postlabeling analysis of bulky DNA adducts was carried out as previously described [13], [14], [15]. Briefly, DNA samples (6 µg) were digested by a mixture of micrococcal endonuclease and spleen phosphodiesterase for 4 h at 37°C. Nuclease P1 was used for adduct enrichment. Labeled DNA adducts were resolved by two-directional thin layer chromatography on 10 cm×10 cm Polygram Cel polyester sheets 300 PEI (Macherey-Nagel, Düren, Germany). Solvent systems used for thin layer chromatography (TLC) were: D-1∶1 M sodium phosphate, pH 6.8; D-2∶3.8M lithium formate, 8.5M urea, pH 3.5; D-3∶0.8 M lithium chloride, 0.5M Tris, 8.5M urea, pH 8.0; D–4 =  D–1. Autoradiography was carried out at −80°C for at least 72 h. Total DNA adduct levels were evaluated from the diagonal radioactive zones (DRZ) on thin layer chromatograms. The DRZ represent the mixture of many overlapping DNA adduct spots originating from the various adduct-forming substances. B[a]P-like DNA adducts were determined using radioactivity detected in the area of chromatograms corresponding to the major B[a]P-derived DNA adduct *N*2-[7,8,9-trihydroxy-7,8,9,10-tetrahydrobenzo[*a*]pyrene-10-yl] deoxyguanosine (BPDE-*N*2-dG) detected in DNA isolated from human peripheral blood mononuclear cells (PBMC) incubated with BPDE (benzo[a]pyrene-r-7,t-8-dihydrodiol-t-9,10-epoxide[±], (from A. Seidel, BIU, Grosshansdorf, Germany) at a 1 µM concentration for 30 min at 37°C. The term B[a]P-like adduct reflects the fact that this adduct spot has the same chromatographic mobility on a TLC sheet as BPDE-N2-dG. However, we cannot exclude or quantify the contribution of other DNA adducts induced by various c-PAHs bound to PM to the amount of B[a]P-like adducts and exhibiting similar chromatographic mobility as BPDE-N2-dG (overlapping with BPDE-N2-dG). The radioactivity of distinct adduct spots and DRZ was measured by liquid scintillation counting. To determine the exact amount of DNA in each sample, aliquots of the DNA enzymatic digest (1 µg of DNA hydrolysate) were analyzed for nucleotide content by reverse-phase HPLC with UV detection, which simultaneously allowed for controlling the purity of the DNA. DNA adduct levels were expressed as adducts per 10^8^ nucleotides. BPDE-*N*2-dG obtained after the incubation of DNA isolated from human PBMC cells (PBMC) with BPDE (see above) was a positive DNA control and was analyzed in each experiment to determine the degree of variability between experiments.

### Levels of XPE, XPC and XPA mRNAs

The cells (3×10^5^) were seeded in 6-well tissue culture plates (TPP, Trasadingen, Switzerland; growth surface 9.0 cm^2^) in 2 ml culture medium supplemented with 10% FBS and incubated for 2–3 days at 37°C. During treatment, the culture medium was replaced with fresh medium supplemented with 1% FBS containing the test chemicals. Each concentration of each sample was analyzed in triplicate. The cells were treated for 6 h, harvested and the cell lysates were stored at −80°C until further processing (max. one week).

RNA was extracted using NucleoSpin RNA II (Macherey-Nagel, Duren, Germany) according to the manufacturer’s instructions; the RNA concentration was measured using a Nanodrop ND-1000 Spectrophotometer (Thermo Fisher Scientific, Waltham, MA, USA). For reverse transcription of total RNA (1000 ng), a Transcriptor High Fidelity cDNA Synthesis Kit (Roche, Mannheim, Germany) was used. Total reaction volume was 20 µl. The reaction was performed according to the manufacurer’s protocol. Quantitative PCR (qPCR) was conducted using a 7900 HT Fast Real-Time PCR System (Applied Biosystems, Carlsbad, CA, USA) as previously described [16]. Raw gene expression data were analyzed with SDS Relative Quantification Software version 2.3 (Applied Biosystems, Carlsbad, CA, USA) to assign the baseline and threshold for Cq determination. Cq values were further analyzed using GenEx software version 5.2.7 (MultiD Analyses AB, Goteborg, Sweden). The expression levels of the target genes were normalized to the expression levels of the reference genes *GAPDH* and *SDHA*. For the gene expression quantification of all genes, we used RealTime Ready Assays (Roche, Mannheim, Germany).

### Levels of XPE, XPC and XPA Proteins

The cells (1×10^6^) were seeded in 75 cm^2^ tissue culture flasks (TPP, Trasadingen, Switzerland) in 15 ml culture medium supplemented with 10% FBS and incubated for 2–3 days at 37°C. For treatment, the culture medium was replaced with 10 ml fresh medium supplemented with 1% FBS containing the test chemicals. The cells were treated for 24 h, harvested and stored at −80°C until further processing.

For Western blotting analysis, the cells were lysed in extraction buffer (1% SDS, 10% glycerol, 100 mM Tris, pH 7.4) supplemented with protease and phosphatase inhibitors, and the protein concentration was estimated using BCA reagent (Sigma, St. Louis, MO, USA). Proteins (12 µg/sample) were separated by SDS-PAGE in 4–12.5% gels (0.75 mm) and transferred onto a Hybond-P PVDF membrane (GE Healthcare, Piscataway, NJ, USA). The membrane was blocked for 2 h at room temperature in TBS buffer (10 mM Tris, 150 mM NaCl, pH 7.4) containing 2% ECL Prime Blocking Agent (GE Healthcare, Piscataway, NJ, USA). The proteins were detected by the incubation of the membrane with primary antibodies against XPE (ab51017; dilution 1∶500), XPC (ab6264; dilution 1∶3000) and XPA (ab2352; 0.1 µg/ml) from Abcam (Cambridge, UK) at 10°C overnight and a secondary antibody conjugated with horseradish peroxidase (NA931; dilution 1∶10 000; GE Healthcare, Piscataway, NJ, USA) at room temperature for 1 h. The antibodies were diluted in TBS-T buffer (TBS +0.1% Tween 20) containing 2% ECL Prime Blocking Agent. To develop the chemiluminiscence signal the membrane was incubated with ECL Prime WB Detection Reagent (GE Healthcare, Piscataway, NJ, USA) for 5 min at room temperature in the dark and exposed to Amersham Hyperfilm (GE Healthcare, Piscataway, NJ, USA). The intensity of the bands on the scanned films was analyzed using ImageJ software (http://rsbweb.nih.gov/ij/index.html). The results were expressed as a percentage of the band intensity of the control sample normalized to a loading control [(the membrane stained with amido black solution (0.1% Amido Black 10B, Sigma-Aldrich, St. Louis, MO, USA; 45% methanol; 10% acetic acid)].

### Unscheduled DNA Synthesis

UDS was measured as the ability of the cells to incorporate 5-ethynyl-2′-deoxyuridine into their DNA after blocking DNA replication with hydroxyurea (HU) [10]. The cells (1.2×10^4^) were seeded in 8-well chamber slides, growth surface 0.8 cm^2^ (Thermo Fisher Scientific, Waltham, MA, USA), in 350 µl culture medium/well supplemented with 10% FBS and incubated for 1–2 days at 37°C. The cells were treated for 24 h with 350 µl of culture medium containing 1% FBS, the test compounds, 5 mM hydroxyurea and 10 µM EdU. This step was followed by an 18-h treatment period in fresh FBS-free medium supplemented with 5 mM hydroxyurea and 10 µM EdU, but without the test chemicals. UDS was detected using the Click-iT EdU Imaging Kit (Invitrogen, Carlsbad, CA, USA) according to the manufacturer’s recommendations, with some modifications. Briefly, the wells were washed with 200 µl PBS and fixed for 5 min in 150 µl ice-cold methanol at −20°C. Methanol was removed and the wells were washed twice with 150 µl 10% FBS in PBS. The slides were then incubated for 30 min with 50 µl of the Click-iT reaction cocktail/well in the dark. Finally, the slides were washed twice with 10% FBS in PBS, air-dried and mounted in Vectashield containing DAPI (1.5 µg/ml). The slides were observed at 200× magnification, and images were recorded using an Axioskop microscope (Zeiss, Jena, Germany) equipped with a CV-M300 CCD camera (JAI, Japan) and ISIS v. 5.0 software (MetaSystems, Altlussheim, Germany). Cells in 20 randomly selected fields per sample were captured, and all photographs were saved for potential future re-analysis. The number of EdU-positive cells among 700–2500 cells was counted, and the percentage of EdU-positive cells relative to the total number of cells was calculated. UDS activity was expressed as a relative value, calculated as the percentage of EdU-positive cells treated with the test compounds divided by the percentage of EdU-positive cells treated with DMSO (a negative control).

### Statistical Analysis

Differences between individual groups in PAH concentrations in EOMs and in the mean values of expression of NER genes and UDS were tested using the two-tailed Student’s t-test; a p-value of 0.05 or less indicated a statistically significant difference between the compared groups. Pearson’s correlation was used to determine associations between the expression levels of NER genes. All calculations were performed using IBM SPSS (Chicago, IL, USA) v. 20 statistical software.

## Results

### Air Sampling and Chemical Analysis of EOMs

Air sampling was conducted in two locations: in Prague, the capital of the Czech Republic, and in Ostrava, an industrial city in Northeastern Moravia characterized by high concentrations of PM2.5 and PAH pollution in the ambient air (Table 1, 2). The samples were collected in February – March 2011 (winter sampling) and in June 2011 (summer sampling). As shown in Table 1, the concentrations of PM2.5 (µg/m^3^) were substantially higher in winter than in summer with the highest level reached in Ostrava. A similar trend was observed for concentrations of B[a]P (ng/m^3^): 1.71, 5.54, 0.02 and 0.41, for Prague-winter, Ostrava-winter, Prague-summer and Ostrava-summer, respectively. The same was true for the yields of EOMs and the concentration of EOM/m^3^ (Table 1). For cell treatment we used equal concentrations of EOMs (1, 10 and 25 µg/ml culture medium) regardless of the season or location. However, chemical analysis of eight selected PAHs revealed that the concentrations of these compounds in EOMs differed (Table 2): total PAH concentrations were significantly higher in the Ostrava-winter than in the Prague-winter EOM and in the Ostrava-summer than in the Prague-summer EOM (p<0.001). We should stress that this data does not reflect differences between the locations and sampling periods expressed in µg EOM/m^3^. When these values were taken into consideration, the observed differences were much greater. Furthermore, it should be noted that the concentrations of PAHs in EOMs used for cell treatment were several orders of magnitude lower than the concentrations of the pure compound (B[a]P) tested in our study. While we analyzed the effects of B[a]P on HEL12469 cells at concentrations of 1, 10 and 25 µM, the corresponding levels of B[a]P in the tested EOMs ranged from 0.4 to 249.6 nM (Table 3).

**Table 1:** Basic characteristics of particulate matter <2.5 µm (PM2.5) samplings.

Sampling location/season	GPS coordinates	Sampling period	Air volume (m^3^)	PM2.5 (µg/m^3^)	EOM (mg)	EOM (µg/m^3^)
Prague-winter	50°0′27.889′′N, 14°26′46.207′′E	February 11– March14 2011	34 863	34.7	118.8	8.50
Ostrava-winter	49°48′52.275′′N, 18°20′23.309′′E	January 7– February 3 2011	39 038	49.2	343.8	22.0
Prague-summer	50°0′27.889′′N, 14°26′46.207′′E	June 1– June 30 2011	50 220	9.2	36.0	1.80
Ostrava-summer	49°48′52.275′′N, 18°20′23.309′′E	June 1– June 30 2011	49 247	16.6	58.1	2.90

**Table 2:** PAH content in extractable organic matter (EOM) from individual samplings.

Analyzed PAHs	Concentrations of PAHs in EOMs from individual sampling locations (ng/µg EOM)			
	Prague-winter*	Ostrava-winter	Prague-summer` *	Ostrava-summer
Benz[a]anthracene	0.22	0.41	0.01	0.11
**Benzo[a]pyrene**	**0.20**	**0.25**	**0.01**	**0.14**
Benzo[b]fluoranthene	0.30	0.41	0.03	0.34
Benzo[g.h.i]perylene	0.16	0.16	0.02	0.17
Benzo[k]fluoranthene	0.13	0.15	0.01	0.13
Chrysene	0.34	0.38	0.02	0.16
Dibenz[a.h]anthracene	0.02	0.03	0.01	0.02
Indeno[1.2.3.cd]pyrene	0.15	0.14	0.02	0.13
Total PAHs	1.51	1.93	0.13	1.20

* Significant differences (p<0.001) in PAH concentrations between EOMs from Prague-summer vs. Ostrava-summer and Prague-winter vs. Ostrava-winter.

**Table 3:** Final concentration of benzo[a]pyrene (B[a]P) in culture media after treatment of the cells with various concentrations of EOM.

	Concentration of EOM in culture media		
	1 µg/ml	10 µg/ml	25 µg/ml
Prague-winter			
B[a]P nM	8.0	79.7	199.1
Ostrava-winter			
B[a]P nM	10.0	99.9	249.6
Prague-summer			
B[a]P nM	0.4	4.4	11.1
Ostrava-summer			
B[a]P nM	5.5	55.2	137.9

### Cell Cultures – growth Curves, Doubling Time and Cytotoxicity

To assess the effect of the test compounds and the S9 fraction on the growth characteristics and viability of HEL12469 cells, we constructed growth curves, calculated the doubling time and assessed cytotoxicity by measuring the activity of mitochondrial succinate-tetrazolium reductases. The analyzed compounds did not significantly affect the growth of cells or their enzymatic activity, indicating that the selected concentrations of chemicals were not cytotoxic under the test conditions. The doubling time of the cells in the presence of the tested compounds increased about 7% (29 h vs. 31 h for control and treated cells, respectively), and cell viability remained about 100% regardless of the treatment conditions.

### Bulky DNA Adducts

B[a]P induced bulky DNA adducts in the HEL12469 cells after their 24 h treatment with the compound, although no clear dose-response was observed. We also found little effect of the S9 fraction on the level of bulky DNA adducts. The effect of EOMs on bulky DNA adduct formation was substantially weaker. While the levels of bulky DNA adducts induced by the winter EOMs were up to 10-fold lower than the levels induced by B[a]P alone, the summer EOMs had minimal effect on DNA damage. Similarly to B[a]P, no clear effect of the presence of the S9 fraction was observed, nor was a dose-response relationship found. However, Ostrava EOMs tended to induce higher DNA adduct levels than EOMs collected in Prague (Table 4).

**Table 4:** Bulky DNA adduct levels/10^8^ nucleotides detected in DNA extracted from HEL12469 cells.

Compound	Concentration	S9 fraction	bulky DNA adducts/10^8^ nucleotides
B[a]P	1 µM	+S9	4.30
B[a]P	10 µM	+S9	9.08
B[a]P	25 µM	+S9	9.96
B[a]P	1 µM	−S9	5.41
B[a]P	10 µM	–S9	8.82
B[a]P	25 µM	–S9	4.46
EOM P-W	1 µg/ml	+S9	0.32
EOM P-W	10 µg/ml	+S9	1.40
EOM P-W	25 µg/ml	+S9	0.56
EOM P-W	1 µg/ml	−S9	0.43
EOM P-W	10 µg/ml	−S9	0.73
EOM P-W	25 µg/ml	−S9	1.74
EOM O-W	1 µg/ml	+S9	N.D.
EOM O-W	10 µg/ml	+S9	2.40
EOM O-W	25 µg/ml	+S9	1.39
EOM O-W	1 µg/ml	−S9	0.78
EOM O-W	10 µg/ml	−S9	0.95
EOM O-W	25 µg/ml	−S9	2.32
EOM P-S	1 µg/ml	+S9	N.D.
EOM P-S	10 µg/ml	+S9	N.D.
EOM P-S	25 µg/ml	+S9	N.D.
EOM P-S	1 µg/ml	−S9	N.D.
EOM P-S	10 µg/ml	−S9	0.10
EOM P-S	25 µg/ml	−S9	N.D.
EOM O-S	1 µg/ml	+S9	N.D.
EOM O-S	10 µg/ml	+S9	N.D.
EOM O-S	25 µg/ml	+S9	1.25
EOM O-S	1 µg/ml	−S9	N.D.
EOM O-S	10 µg/ml	−S9	N.D.
EOM O-S	25 µg/ml	−S9	0.34

The cells were treated for 24 h with benzo[a]pyrene (B[a]P) and extractable organic matter (EOM) in the absence (–S9) and presence (+S9) of the microsomal S9 fraction. P–W = Prague-winter, O–W = Ostrava-winter, P–S = Prague-summer, O–S = Ostrava-summer, N.D. – not detectable.

### The Effect of B[a]P and EOMs on XPE, XPC and XPA mRNA Levels

**Figure 1:**
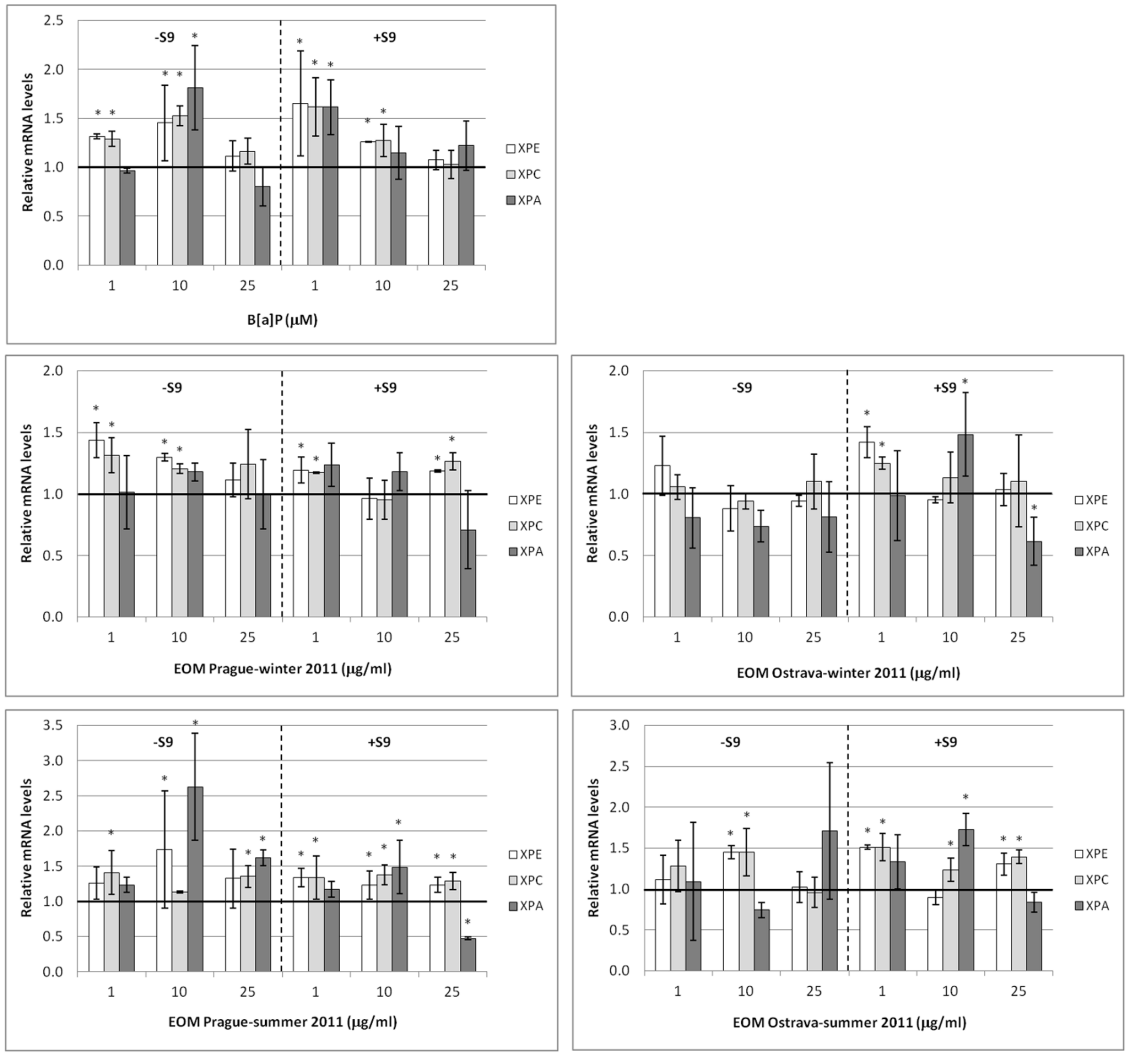
Relative levels of XPE, XPC and XPA mRNAs. The levels of mRNAs were analyzed after the 6 h treatment of HEL12469 cells with benzo[a]pyrene (B[a]P) and extractable organic matter (EOM) in the absence (–S9) and presence (+S9) of the microsomal S9 fraction. Mean ± SD values from three independent cell treatments are shown, asterisks denote a significant (p<0.05) increase/decrease of mRNA levels. The baseline mRNA level after treatment of the cells with DMSO is represented by a bold horizontal line.

The results are presented in Figure 1. The cells were treated with the tested compounds for 6 h. In general, we observed changes in XPE and XPC mRNA levels; the induction of changes in XPA mRNA levels was less pronounced.

Exposure to lower concentrations of B[a]P (1 and 10 µM) both in the presence and absence of the S9 fraction caused a significant increase of XPE and XPC mRNA levels; this was not observed when the cells were treated with 25 µM B[a]P. The effect of B[a]P on XPA mRNA levels was weak.

The EOMs collected in the winter season, particularly the Ostrava EOMs, were weaker inducers of mRNA levels when compared with the summer EOMs. While the Prague-winter EOMs affected XPE and XPC mRNA levels, almost no effect of the Ostrava-winter EOMs on mRNA levels was observed. There was no difference between samples treated in the presence and absence of the S9 microsomal fraction. The treatment of the cells with the summer EOMs in the presence of the S9 microsomal fraction resulted in the induction of XPE and XPC mRNA levels. The effect of the –S9 summer EOMs was weaker, particularly for the samples collected in Ostrava.

We found a significant positive correlation between XPE and XPC mRNA levels (R = 0.761, p<0.001 and R = 0.436, p<0.001 for samples treated with B[a]P and EOMs, respectively), as well as between XPC and XPA mRNA levels in samples treated with B[a]P (R = 0.613, p<0.01).

### XPE, XPC and XPA Protein Levels

**Figure 2:**
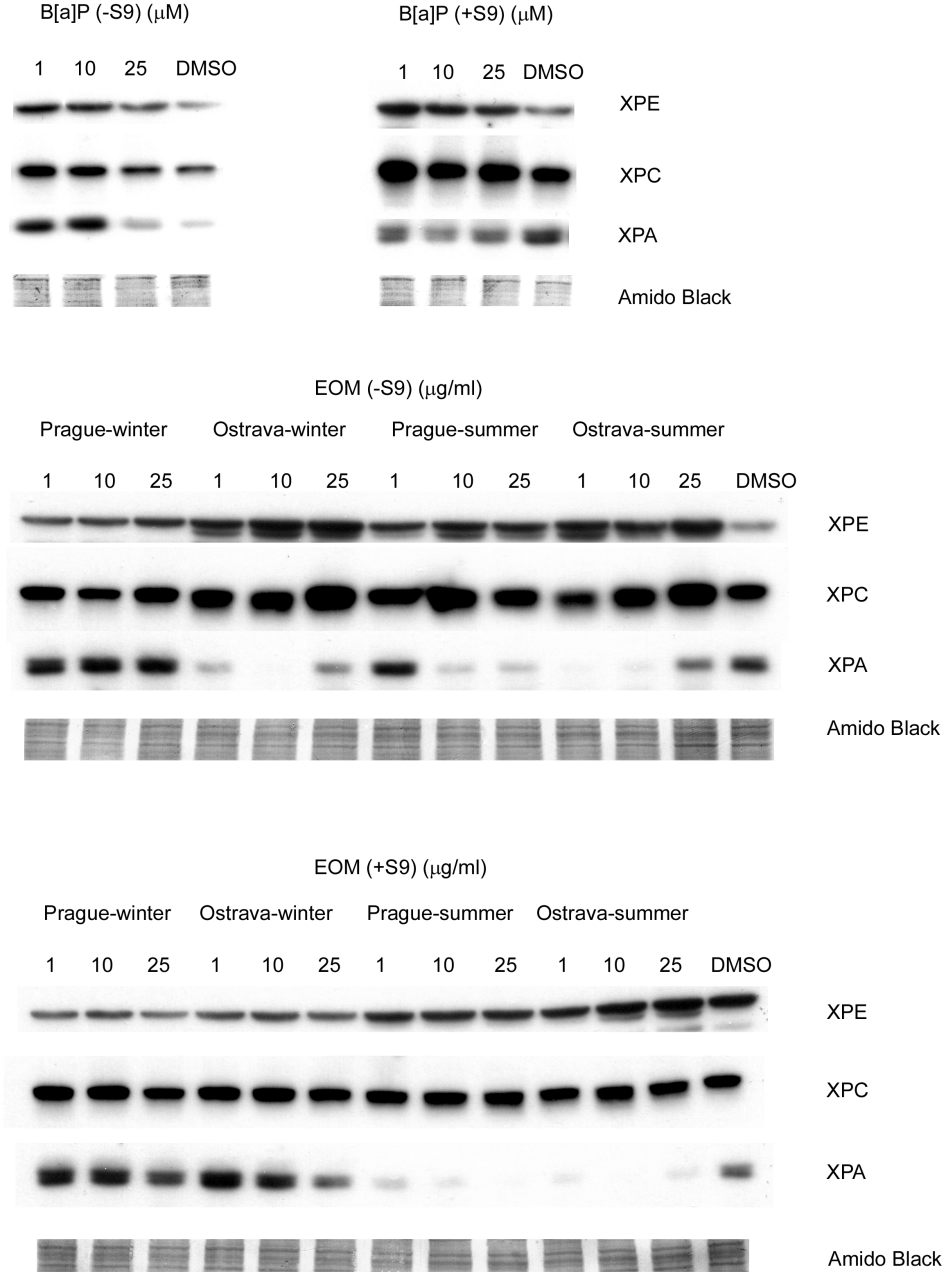
Western blotting analyses of the levels of XPE, XPC and XPA proteins. Protein expression was measured after the 24 h treatment of HEL12469 cells with benzo[a]pyrene (B[a]P) and extractable organic matter (EOM) in the absence (–S9) and presence (+S9) of the microsomal S9 fraction. A representative result of two independent experiments is shown. Amido Black-stained proteins were used as a loading control.

The effect of 24 h treatment with the tested compounds on NER protein levels was different for B[a]P and the EOMs. We observed further differences between samples treated in the presence and absence of the S9 microsomal fraction and the winter and summer EOMs (Fig. 2, Table 5).

**Table 5:** Relative levels of XPE, XPC and XPA proteins in lysates of HEL12469 cells.

			Relative protein levels (%; control = 100%)		
Compound	Concentration	S9 fraction	XPE	XPC	XPA
B[a]P	1 µM	+	162	89	47
B[a]P	10 µM	+	130	70	34
B[a]P	25 µM	+	117	81	48
B[a]P	1 µM	−	478	246	1437
B[a]P	10 µM	−	421	218	1644
B[a]P	25 µM	−	350	191	450
EOM P-W	1 µg/ml	+	32	91	167
EOM P-W	10 µg/ml	+	47	120	221
EOM P-W	25 µg/ml	+	33	97	159
EOM P-W	1 µg/ml	−	158	112	150
EOM P-W	10 µg/ml	−	167	91	155
EOM P-W	25 µg/ml	−	202	116	150
EOM O-W	1 µg/ml	+	44	104	224
EOM O-W	10 µg/ml	+	46	99	163
EOM O-W	25 µg/ml	+	42	96	92
EOM O-W	1 µg/ml	−	303	132	33
EOM O-W	10 µg/ml	−	415	156	5
EOM O-W	25 µg/ml	−	355	169	51
EOM P-S	1 µg/ml	+	68	102	25
EOM P-S	10 µg/ml	+	71	106	11
EOM P-S	25 µg/ml	+	81	117	4
EOM P-S	1 µg/ml	−	229	133	126
EOM P-S	10 µg/ml	−	306	170	22
EOM P-S	25 µg/ml	−	297	137	27
EOM O-S	1 µg/ml	+	65	87	6
EOM O-S	10 µg/ml	+	104	108	3
EOM O-S	25 µg/ml	+	132	116	14
EOM O-S	1 µg/ml	−	355	113	6
EOM O-S	10 µg/ml	−	323	155	10
EOM O-S	25 µg/ml	−	325	159	71

The cells were treated with B[a]P and EOMs for 24 h. The data represent mean protein levels relative to the control sample from two independent experiments. P–W = Prague-winter, O–W = Ostrava-winter, P–S = Prague-summer, O–S = Ostrava-summer.

Lysates of B[a]P-treated cells incubated in the absence of the S9 microsomal fraction showed elevated levels of the three analyzed proteins, but the results suggested a negative dose-response relationship. While adding the S9 microsomal fraction to the growth medium had no effect on the levels of XPC, it increased the levels of XPE and decreased the levels of XPA.

Treatment with all of the tested EOMs in the absence of the S9 microsomal fraction increased the levels of the XPE protein. A similar, but less pronounced, effect was observed for the XPC protein. The XPA protein levels were elevated after treatment with the +S9 winter EOMs; all summer samples inhibited the expression of this protein.

Correlation analysis showed significant positive associations between the protein levels of XPE and XPC (R = 0.997, p<0.001 and R = 0.827, p<0.001 for samples treated with B[a]P and EOMs, respectively) and between XPE and XPA levels for samples treated with B[a]P (R = 0.920, p<0.01) and a negative correlation between XPE and XPA levels for samples treated with EOM (R = −0.455, p<0.05).

### Unscheduled DNA Synthesis

**Figure 3:**
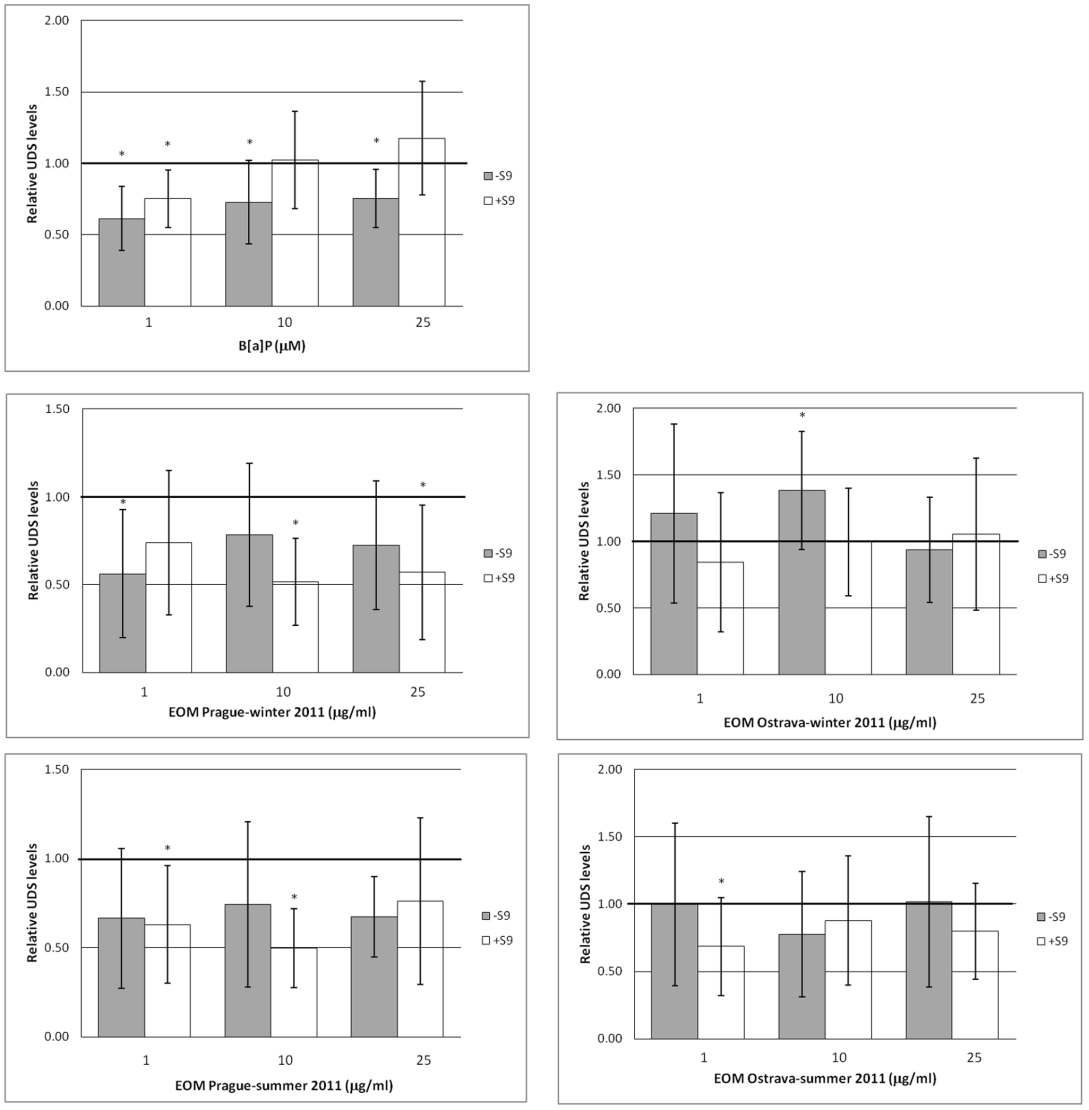
Relative levels of unscheduled DNA synthesis (UDS). UDS in HEL12469 cells was studied after 24 h treatment with benzo[a]pyrene (B[a]P) and extractable organic matter (EOMs) in the absence (–S9) and presence (+S9) of the S9 microsomal fraction. Mean ± SD UDS values relative to the DMSO-treated control are shown, asterisks denote a significant (p<0.05) increase/decrease in the activity of UDS. The baseline levels of UDS in cells treated with DMSO is represented by a bold horizontal line. Each mean UDS value is based on the analysis of 700–2500 cells.

We first compared the relative UDS levels after the 24 h exposure of the cells to B[a]P, separately for the +S9 and –S9 samples (Figure 3). UDS levels in the –S9 samples were significantly lower than in controls regardless of the concentration of B[a]P. For the +S9 samples, a significant decrease in UDS activity was found only in the sample treated with the lowest concentration of B[a]P (1 µM). Interestingly, UDS activity was significantly higher in the +S9 than in –S9 samples for all B[a]P concentrations tested (p = 0.045, p<0.01 and p<0.001 for 1, 10 and 25 µM B[a]P).

The treatment of the cells with EOMs resulted mostly in a drop in the UDS activity with the exception of the –S9 Ostrava-winter EOM, concentration 10 µg/ml. We observed no difference between UDS levels measured after the treatment of the cells with the winter and summer EOMs. In general, UDS induced by the Ostrava EOMs was higher than the UDS induced by EOMs collected in Prague.

## Discussion

The purpose of our study was to analyze the effect of PAHs (B[a]P as a pure compound and complex mixtures of PAHs in EOMs) on DNA damage and the DNA repair response in HEL12469 cells. We selected a normal, non-tumor cell line in order to better mimic the conditions in human lungs exposed to air pollutants. In the past we used human embryonic lung fibroblasts (HEL cells) from a local supplier (Sevapharma, Prague, Czech Republic) and analyzed various parameters, including the expression of p53, p21/WAF1, hsp32 and hsp70 proteins, as well as bulky DNA adduct levels [12], [17], [18], [19], [20]. We proved that in this cell line PAHs are successfully metabolized and bulky DNA adducts are formed (the levels exceeding 100 adducts/10^8^ nucleotides, [12]). Due to the fact that HEL cells are no longer available from the original source, we obtained HEL12469 cells from the ECACC because their characteristics were identical. For this reason, we also used the same treatment conditions (concentrations of tested compounds, time of treatment) as in our previous studies. As HEL cells have been shown to exhibit lower metabolic activity for PAHs and to form lower levels of bulky DNA adducts when compared with hepatocytes [20], we opted for the use of the rat microsomal S9 fraction that contains liver metabolic enzymes to improve the response of the cells to treatment with B[a]P and EOMs. We hypothesized that in the samples treated with the S9 fraction, the end points examined would be more pronounced. Also, the different sources of air pollution and the concentrations of pollutants in the ambient air of the two Czech cities may impact the effect of EOMs on the analyzed markers, even though we used equal concentrations of these compounds for the treatment of the cells.

We analyzed mRNA levels after 6 h treatment with the tested compounds, while the other endpoints (protein levels, UDS and DNA adduct levels) were measured after 24 h treatment. It is estimated that differences in protein concentrations are only 20–40% attributable to changes in mRNA levels, which underlines the importance of post-translation modifications of proteins [21]. Since changes in mRNA levels represent an early event in the cellular response to DNA damaging agents, while achieving steady-state levels of proteins, including their post-translation modifications, is a longer process [22], we analyzed mRNA and protein levels at different time points.

B[a]P and to lesser extent winter EOMs induced bulky DNA adduct formation. The effect of summer EOMs regardless of the location was minimal. In general, the levels of DNA adducts were very low and no clear dose-response was detected at selected concentrations (for B[a]P: 1, 10 and 25 µM). We therefore tested the effect of lower concentrations of B[a]P (0.01 µM and 0.1 µM) on bulky DNA adduct induction. The data show that at the lowest tested dose, bulky DNA adduct levels were only 1.78 adduct/10^8^ nucleotides. However, after increasing the concentration to 0.1 µM B[a]P, bulky DNA adduct levels seemed to reach a plateau and did not substantially elevate with higher B[a]P concentrations. Since the B[a]P doses of 0.01 and 0.1 µM had no effect on other tested endpoints (data not shown), we used higher doses of B[a]P for our experiments. Moreover, in our previous studies with various cell lines including human embryonic lung fibroblasts obtained from a different source than the cells in the present study (HEL cells) [12], [19], [20], the levels of bulky DNA adducts induced by B[a]P (1 and 10 µM) were at least 10-fold higher than those observed in the present study. Since the treatment of the cells with B[a]P in the presence of the S9 microsomal fraction had no major effect on bulky DNA adduct formation, we speculate that the low levels of DNA adducts in HEL12469 cells are only partially caused by the low metabolic activity of the cells. It seems probable that other cellular processes also play a role, but they are currently unknown and should be identified in future studies. Low levels of bulky DNA adducts suggest that HEL12469 cells may not be an optimal model for *in vitro* testing of DNA damage by carcinogens.

NER consists of three steps: pre-incision, incision and post-incision [23]. In the pre-incision step, XPC is a crucial protein which recognizes helix distortion in DNA and thus initiates NER [24]. XPE (DDB2), a part of the UV-DDB complex, is a key molecule responsible for the detection of UV-induced DNA damage [25], but it also recognizes other lesions in DNA thus acting as a sensor searching DNA for conformational changes [26], [27]. XPE recruits XPC to DNA lesions, after which both proteins are ubiquitylated; the ubiquitylation of XPE leads to its degradation, while the ubiquitylation of XPC increases its affinity for DNA [25]. XPE also interacts with DNA lesions independently of XPC, and there is little interaction between the two proteins on damaged DNA [25]. XPA plays a role in the verification of DNA damage. It binds to the unwound DNA molecule along with the RPA protein [28], [29].

After treatment of HEL12469 cells with B[a]P, we observed an increase in the expression of both the *XPE* and *XPC* genes, but only when lower concentrations of the compound (1 and 10 µM) were used. A negative association between mRNA levels and the B[a]P concentrations used could be theoretically explained by the toxicity of higher doses of B[a]P. However, neither cell growth nor the mitochondrial dehydrogenase activity of the cells differed after treatment with the tested concentrations of B[a]P. We may speculate that B[a]P caused negative changes in the cells that were not revealed by our cytotoxicity tests, but may have been manifested by a decrease in the expression of the NER genes. We also observed a very close correlation between the levels of XPE and XPC mRNAs, which is in agreement with the fact that both genes are regulated in a similar manner and their protein products participate in the same phase of NER [30].

The EOMs collected in Prague, particularly those collected in the winter season, induced a similar pattern of the *XPE* and *XPC* gene expression as B[a]P, although the expression levels were mostly lower. A similar observation was made for the Ostrava summer EOM. In contrast, the effect of the winter Ostrava EOMs on the expression of both genes was substantially weaker.

The pattern of XPA mRNA levels following both B[a]P and EOM treatment differed from that of the *XPE* and *XPC* genes, and no significant dose-response relationship was observed. The EOMs collected in summer in Prague, i.e. the samples containing the lowest concentrations of PAHs, were the strongest inducers of *XPA* expression. Interestingly, we noted a significant correlation between XPA and XPC levels, which may support the observation that the protein products of both genes interact when XPA binds to DNA [29].

Treatment of the HEL12469 cells with B[a]P in the absence of the S9 fraction had the strongest effect on the levels of the XPE, XPC and XPA proteins, while +S9 samples exhibited a substantially weaker response. This result may be explained by a faster accumulation of the reactive products of B[a]P metabolism due to the presence of the microsomal fraction in the +S9 samples. Consequently, after 24 h treatment, the concentrations of the analyzed proteins may have already dropped to their basal levels. In a recent study, it was shown that the levels of NER proteins, including XPC and XPA, change depending on the concentrations of B[a]P and the length of treatment [31]. Although the authors observed increased levels of both proteins, their response was not consistently related to the tested concentrations of B[a]P (2, 8 and 16 µmol/l). Although the response of the cells after EOM treatment was also mostly weaker after +S9 treatment, it differed from B[a]P treatment in several aspects. Both XPE and XPC expression was induced in the –S9 samples regardless of the location or season; the expression levels were about half of those observed for B[a]P. XPA expression, which was very high after B[a]P treatment, was inhibited in EOM-treated –S9 samples. Further, winter EOM-treatment in the presence of the S9 fraction increased XPA expression; this effect was not observed for B[a]P treatment.

We expected that elevated levels of the examined NER proteins would be accompanied by increased UDS activity. To test this assumption, we used a recently introduced modification of the UDS assay [9], [10]. Our results showed that 24 h B[a]P treatment had either no effect or inhibited UDS activity; the inhibition was more pronounced in the absence of the S9 fraction. Treatment with the EOMs had a similar effect on UDS activity with no apparent differences between seasons. The effects of Ostrava EOMs were more pronounced when compared with both Prague EOMs and B[a]P; however, a significant increase in UDS activity was observed for only one tested concentration.

As it has been shown that reactive metabolites of B[a]P induce UDS [32], we expected increased UDS activity particularly in +S9 samples. NER may be affected both on the level of transcription regulation and also the activity of proteins. It has been shown that the expression of the *XPC* and *XPE* genes is regulated by p53, which acts as a transcription factor inducing NER in response to DNA damage [30], [33]. We previously reported that B[a]P induces the expression of p53 in HEL cells [12]. Although the p53 response to treatment was not analyzed in HEL12469 cells, levels of both XPC and XPE mRNAs were induced. Thus, inhibition of the activity of NER proteins is a more probable scenario. According to studies by Feng et al. [34], [35], malondialdehyde (MDA) and trans-4-hydroxy-nonenal (4-HNE), products of lipid peroxidation, inhibited NER in cells treated with BPDE by interaction with cellular repair proteins. Although B[a]P may induce oxidative stress, our previous report showed that B[a]P treatment had a weak effect on lipid peroxidation, assessed as 15-F_2t_-isoprostane (15-F2t-IsoP) levels, in HEL cells [11]. However, we did not use the S9 fraction to facilitate B[a]P activation and did not analyze MDA and 4-HNE levels, which may differ from 15-F2t-IsoP levels. Thus, it is difficult to draw conclusions based on the data obtained from this cell line. Other experiments that are beyond the scope of the present study would be required in the future.

It has been further reported that EOMs obtained from air particulate matter collected at different locations in winter and summer induced UDS in primary rat hepatocytes [36]. The authors observed a dose-response relationship between EOM concentration and UDS, but no difference by locality or season. However, the study did not report the size of the collected PM or information on the chemical analysis of the EOMs, which may significantly affect the results and may explain the discrepancies with our data.

Although the genotoxic effects of the EOMs tested in our study were generally weak when compared to B[a]P alone, we observed higher bulky DNA adduct levels in samples treated with winter EOMs, with slightly higher values for the Ostrava samples. This observation, along with the effect of B[a]P tested in concentrations about 100-fold higher than the concentration of the sum of eight analyzed PAHs in EOMs, confirms that PAH content is a factor responsible for the genotoxicity of the tested compounds. However, other endpoints do not seem to depend strictly on the PAH concentrations in the tested compounds. Ostrava winter EOMs, containing the highest levels of PAHs, had no effect on mRNA levels, while other EOMs, including Prague summer EOMs, affected mRNA expression. The same was true for B[a]P treatment. Most of the NER proteins responded in a similar way to EOM treatment regardless of PAH content with generally low levels of XPA expression. In contrast, B[a]P induced the very high expression of XPA. Finally, UDS does not seem to be affected by either PAH content in EOMs or by any B[a]P concentration tested. As evident from a detailed chemical analysis of EOMs from PM2.5 collected in Ostrava performed in our recent study [37], EOMs contain a complex mixture of other compounds including other PAHs and their derivatives (e.g., alkylated, nitrated or oxidized PAHs), n-alkanes, sterols and various industrial contaminants. These compounds were not assessed in the EOMs used in the present study; however, it is very probable that EOMs from different places collected in different seasons differ in the content of these chemicals. Moreover, it has been shown that the presence of other compounds in EOMs may have an inhibitory effect on B[a]P-DNA adduct formation [19]. These compounds may induce other forms of DNA damage, affect the analyzed endpoints and thus modify the response of the cells to the tested EOMs. Taken together, the content of the analyzed PAHs itself is probably not a key factor affecting NER repair processes in our study. Other unidentified compound(s) (or interactions between them) may be responsible for the NER response after EOM treatment.

In conclusion, we have shown that most of the tested samples induced bulky DNA adduct formation, although their levels were lower than expected. We also observed the ability of HEL12469 cells to respond to treatment with B[a]P and EOMs by the induction of the expression of selected NER genes, both on the level of mRNA and proteins. The response was partially affected by the presence of the S9 microsomal fraction as well as by the location and season of EOM collection. However, the expression of NER genes was not reflected in the activity of DNA repair. The qualitatively different results observed for B[a]P and various EOMs in this study suggest that many EOM components other than B[a]P and PAHs likely play a significant role in the DNA damage response in HEL12469 cells. At least some of these components remain to be identified by future studies based on a more detailed chemical characterization of EOM coupled with an analysis of the complex DNA damage response in relevant human cell lines.

## References

[1] Schlesinger RB (2007) The health impact of common inorganic components of fine particulate matter (PM2.5) in ambient air: a critical review. Inhal Toxicol 19: 811–832. 17687714 10.1080/08958370701402382

[2] Valavanidis A, Fiotakis K, Vlachogianni T (2008) Airborne particulate matter and human health: toxicological assessment and importance of size and composition of particles for oxidative damage and carcinogenic mechanisms. J Environ Sci Health C Environ Carcinog Ecotoxicol Rev 26: 339–362. 19034792 10.1080/10590500802494538

[3] Pelucchi C, Negri E, Gallus S, Boffetta P, Tramacere I, et al (2009) Long-term particulate matter exposure and mortality: a review of European epidemiological studies. BMC Public Health 9: 453. 19995424 10.1186/1471-2458-9-453 PMC2797801

[4] Harrison RM, Smith DJ, Kibble AJ (2004) What is responsible for the carcinogenicity of PM2.5? Occup Environ Med 61: 799–805. 15377764 10.1136/oem.2003.010504 PMC1740668

[5] Xue W, Warshawsky D (2005) Metabolic activation of polycyclic and heterocyclic aromatic hydrocarbons and DNA damage: a review. Toxicol Appl Pharmacol 206: 73–93. 15963346 10.1016/j.taap.2004.11.006

[6] IARC (2012) IARC monographs on the Evaluation of the Carcinogenic Risk of Chemicals to Humans. Chemical Agents and Related Occupations. Lyon, France: IARC Publications. PMC4781612 23189753

[7] Baird WM, Hooven LA, Mahadevan B (2005) Carcinogenic polycyclic aromatic hydrocarbon-DNA adducts and mechanism of action. Environ Mol Mutagen 45: 106–114. 15688365 10.1002/em.20095

[8] Michalopoulos G, Sattler GL, O’Connor L, Pitot HC (1978) Unscheduled DNA synthesis induced by procarcinogens in suspensions and primary cultures of hepatocytes on collagen membranes. Cancer Res 38: 1866–1871. 666894

[9] Limsirichaikul S, Niimi A, Fawcett H, Lehmann A, Yamashita S, et al (2009) A rapid non-radioactive technique for measurement of repair synthesis in primary human fibroblasts by incorporation of ethynyl deoxyuridine (EdU). Nucleic Acids Res 37: e31. 19179371 10.1093/nar/gkp023 PMC2651789

[10] Salic A, Mitchison TJ (2008) A chemical method for fast and sensitive detection of DNA synthesis in vivo. Proc Natl Acad Sci U S A 105: 2415–2420. 18272492 10.1073/pnas.0712168105 PMC2268151

[11] Hanzalova K, Rossner P Jr, Sram RJ (2010) Oxidative damage induced by carcinogenic polycyclic aromatic hydrocarbons and organic extracts from urban air particulate matter. Mutat Res 696: 114–121. 20079458 10.1016/j.mrgentox.2009.12.018

[12] Binkova B, Giguere Y, Rossner P Jr, Dostal M, Sram RJ (2000) The effect of dibenzo[a,l]pyrene and benzo[a]pyrene on human diploid lung fibroblasts: the induction of DNA adducts, expression of p53 and p21(WAF1) proteins and cell cycle distribution. Mutat Res 471: 57–70. 11080661 10.1016/s1383-5718(00)00111-x

[13] Binkova B, Chvatalova I, Lnenickova Z, Milcova A, Tulupova E, et al (2007) PAH-DNA adducts in environmentally exposed population in relation to metabolic and DNA repair gene polymorphisms. Mutat Res 620: 49–61. 17412371 10.1016/j.mrfmmm.2007.02.022

[14] Phillips DH, Castegnaro M (1999) Standardization and validation of DNA adduct postlabelling methods: report of interlaboratory trials and production of recommended protocols. Mutagenesis 14: 301–315. 10374998 10.1093/mutage/14.3.301

[15] Reddy MV, Randerath K (1986) Nuclease P1-mediated enhancement of sensitivity of [32P]- postlabeling test for structurally diverse DNA adducts. Carcinogenesis 7: 1543–1551. 3017601 10.1093/carcin/7.9.1543

[16] Rossner P Jr, Uhlirova K, Beskid O, Rossnerova A, Svecova V, et al (2011) Expression of XRCC5 in peripheral blood lymphocytes is upregulated in subjects from a heavily polluted region in the Czech Republic. Mutat Res 713: 76–82. 21684294 10.1016/j.mrfmmm.2011.06.001

[17] Rossner P Jr, Binkova B, Chvatalova I, Sram RJ (2002) Acrylonitrile exposure: the effect on p53 and p21(WAF1) protein levels in the blood plasma of occupationally exposed workers and in vitro in human diploid lung fibroblasts. MutatRes 517: 239–250. 10.1016/s1383-5718(02)00081-5 12034325

[18] Rossner P Jr, Binkova B, Sram RJ (2003) Heat shock proteins hsp32 and hsp70 as biomarkers of an early response? In vitro induction of heat shock proteins after exposure of cell culture to carcinogenic compounds and their real mixtures. Mutat Res 542: 105–116. 14644359

[19] Binkova B, Sram RJ (2004) The genotoxic effect of carcinogenic PAHs, their artificial and environmental mixtures (EOM) on human diploid lung fibroblasts. Mutat Res 547: 109–121. 15013705 10.1016/j.mrfmmm.2003.12.006

[20] Sevastyanova O, Binkova B, Topinka J, Sram RJ, Kalina I, et al (2007) In vitro genotoxicity of PAH mixtures and organic extract from urban air particles part II: human cell lines. Mutat Res 620: 123–134. 17420030 10.1016/j.mrfmmm.2007.03.002

[21] Brockmann R, Beyer A, Heinisch JJ, Wilhelm T (2007) Posttranscriptional expression regulation: what determines translation rates? PLoS Comput Biol 3: e57. 17381238 10.1371/journal.pcbi.0030057 PMC1829480

[22] Hargrove JL, Hulsey MG, Beale EG (1991) The kinetics of mammalian gene expression. BioEssays 13: 667–674. 1789784 10.1002/bies.950131209

[23] Lagerwerf S, Vrouwe MG, Overmeer RM, Fousteri MI, Mullenders LH (2011) DNA damage response and transcription. DNA repair 10: 743–750. 21622031 10.1016/j.dnarep.2011.04.024

[24] Volker M, Mone MJ, Karmakar P, van Hoffen A, Schul W, et al (2001) Sequential assembly of the nucleotide excision repair factors in vivo. Mol Cell 8: 213–224. 11511374 10.1016/s1097-2765(01)00281-7

[25] Luijsterburg MS, Goedhart J, Moser J, Kool H, Geverts B, et al (2007) Dynamic in vivo interaction of DDB2 E3 ubiquitin ligase with UV-damaged DNA is independent of damage-recognition protein XPC. J Cell Sci 120: 2706–2716. 17635991 10.1242/jcs.008367

[26] Payne A, Chu G (1994) Xeroderma pigmentosum group E binding factor recognizes a broad spectrum of DNA damage. Mutat Res 310: 89–102. 7523888 10.1016/0027-5107(94)90012-4

[27] Wittschieben BO, Iwai S, Wood RD (2005) DDB1-DDB2 (xeroderma pigmentosum group E) protein complex recognizes a cyclobutane pyrimidine dimer, mismatches, apurinic/apyrimidinic sites, and compound lesions in DNA. J Biol Chem 280: 39982–39989. 16223728 10.1074/jbc.M507854200

[28] Croteau DL, Peng Y, Van Houten B (2008) DNA repair gets physical: mapping an XPA-binding site on ERCC1. DNA Repair (Amst) 7: 819–826. 18343204 10.1016/j.dnarep.2008.01.018 PMC2494945

[29] Shell SM, Zou Y (2008) Other proteins interacting with XP proteins. Adv Exp Mol Biol 637: 103–112. 10.1007/978-0-387-09599-8_11 PMC3117267 19181115

[30] Sugasawa K (2010) Regulation of damage recognition in mammalian global genomic nucleotide excision repair. Mutat Res 685: 29–37. 19682467 10.1016/j.mrfmmm.2009.08.004

[31] Yang J, Liu X, Niu P, Zou Y, Gong Z, et al (2007) Dynamic changes of XPA, XPC, XPF, XPG and ERCC1 protein expression and their correlations with levels of DNA damage in human bronchial epithelia cells exposed to benzo[a]pyrene. Toxicol Lett 174: 10–17. 17900831 10.1016/j.toxlet.2007.08.004

[32] Chen JK, Wu ZL, Liu YG, Lei YX (2000) Effects of metabolites of benzo(a)pyrene on unschedule DNA synthesis in BALB/3T3 cell line. Chemosphere 41: 139–142. 10819191 10.1016/s0045-6535(99)00401-4

[33] Adimoolam S, Ford JM (2003) p53 and regulation of DNA damage recognition during nucleotide excision repair. DNA Repair (Amst) 2: 947–954. 12967652 10.1016/s1568-7864(03)00087-9

[34] Feng Z, Hu W, Tang MS (2004) Trans-4-hydroxy-2-nonenal inhibits nucleotide excision repair in human cells: a possible mechanism for lipid peroxidation-induced carcinogenesis. Proc Natl Acad Sci U S A 101: 8598–8602. 15187227 10.1073/pnas.0402794101 PMC423240

[35] Feng Z, Hu W, Marnett LJ, Tang MS (2006) Malondialdehyde, a major endogenous lipid peroxidation product, sensitizes human cells to UV- and BPDE-induced killing and mutagenesis through inhibition of nucleotide excision repair. Mutat Res 601: 125–136. 16872641 10.1016/j.mrfmmm.2006.06.003

[36] Zhao X, Wan Z, Chen G, Zhu H, Jiang S, et al (2002) Genotoxic activity of extractable organic matter from urban airborne particles in Shanghai, China. Mutat Res 514: 177–192. 11815256 10.1016/s1383-5718(01)00338-2

[37] Libalova H, Uhlirova K, Klema J, Machala M, Sram RJ, et al (2012) Global gene expression changes in human embryonic lung fibroblasts induced by organic extracts from respirable air particles. Part Fibre Toxicol 9: 1. 22239852 10.1186/1743-8977-9-1 PMC3275518

